# Geographical origin discrimination of Ethiopian sesame seeds by elemental analysis and chemometric tools

**DOI:** 10.1016/j.fochx.2022.100545

**Published:** 2022-12-13

**Authors:** Wasihun Abebe Hika, Minaleshewa Atlabachew, Meareg Amare

**Affiliations:** Department of Chemistry, College of Science, Bahir Dar University, Bahir Dar 79, Ethiopia

**Keywords:** Sesame seed, Geographical origin discrimination, Multi-element analysis, Chemometric tools, ICP-OES, PCA, LDA

## Abstract

Origin discrimination of sesame seeds is becoming one of the important factors for the sesame seed trade in Ethiopia as it influences the market price. This study was undertaken to construct accurate geographical origin discriminant models for Ethiopian sesame seeds using multi-element analysis and statistical tools. The concentration of 12 elements (Na, Mg, Cr, Mn, Fe, Cu, Co, Ni, Zn, Cd, As and Pb) were determined in 93 samples which were collected from three main sesame seed-producing regions in Ethiopia, Gondar, Humera and Wollega. According to a one-way analysis of variance (ANOVA), the concentration of 10 elements showing a significant difference (p < 0.05) was taken for statistical analysis using principal component analysis (PCA) and linear discriminant analysis (LDA). PCA showed some clustering of samples according to their respective origins. Then, the follow-up LDA resulted in a 100 % correct origin classification rate for all 93 sesame seed samples obtained from three regions in Ethiopia.

## Highlights

• PCA model shows some clustering of samples according to their respective origins.
• LDA is very successful to discriminate the three sesame seed varieties originated in Ethiopia.
• Multi-element analysis & statistical tools are effective way to discriminate sesame origins.

## 1 Introduction

For the past few years, geographical origin authentication of food products has been gaining a growing concern among consumers, retailers, farmers and food authorities. Agricultural food producers and manufacturers have introduced geographical indications (GIs) to protect products in a particular region which is considered the best and most distinctive by consumers (UNFAO, 2015). Origin-based agricultural products are highly favored for their specific culinary organoleptic qualities or features resulting from a unique combination of natural resources (such as climatic conditions, soil characteristics and local plant varieties), traditional local skills and knowledge, historical and cultural practices and patriotism. Other influencing reasons are a lower confidence in the quality and safety of products produced outside their local region or country, concern about human and animal welfare resulting from environmentally unfriendly production methods and may be media coverage (Dion et al., 2008**).** GIs hence, give products their specific characteristics, qualities or reputations resulting from their geographical origin. It also gives recognition and intellectual property rights and, therefore, offers both a helpful marketing tool and protection of the ownership of products. In 1992, European Union (EU) regulations 2081/92 and 2082/92 introduced an integrated framework for the protection of geographical indications and designations of origin for agricultural food products. The regulation also seeks to achieve wider social and environmental objectives with respect to the rural economy.

Sesame (*Sesamum indicum L. Pedaliaceae*) is one of the oldest agricultural products thought to have originated in Africa (Bedigian et al., 1985). Sesame seed contains approximately 40 %–60 % oil, which is mainly composed of mono- and polyunsaturated fatty acids, accounting for almost 85 % of the total fatty acids. The high oil quality and its benefit to human health have earned the seed a poetic label “queen of oilseed” (Agidew et al., 2021).

Because of the popularity of sesame oil among consumers, the market demand for the seed is growing rapidly, and the planting area of sesame has almost doubled in the last fifty. Ethiopia is one of the largest sesame seed producer (UNFAO, 2015) in the world. The major sesame producing regions are located in the North and South-west Ethiopian in Gondar, Humera, and Wollega. These three areas contribute more than 92 % of the total sesame output in the country (Zerihun, 2012). The seed production in the country is highly growing due to its high market value and suitability to environmental conditions. The quality of sesame varieties in Ethiopia is usually known by their commercial brand name, such as, Humera, Gondar and Wollega types. The Humera type is the most worldwide appreciated sesame seed for its aroma and sweet taste. Despite the high potential for increased production and the rapidly growing demand in the international market for Ethiopian sesame, the supply chain of the seed is being highly suffered mainly due to quality and market price differences for various seeds originated from various region in the country (Zerihun, 2012). This causes fraudulent stakeholders not to be transparent, hence, misleading the origin of the seeds. Thus, it is crucial to select and grade sesame seeds according to their quality and clearly specify its characteristics, such as its origin (for traceability), organoleptic qualities or a specialty. This creates good price, enhanced market access, better market competition and distribution and gives recognition of the role of primary producers. It also helps to protect regional brands and enhance consumers’ confidence. Accordingly, a healthy, strong supply chain of sesame seeds is established from the local producers in the country to users of the seeds in the world.

Due to the differences in hydrological characteristics and geological background, different agricultural food products have different element profiles, which provide the possibility of geographical traceability for the given product (Zhang, et al., 2020). Hence, mineral content determination and chemometric pattern recognition have been among the most popular analytical techniques employed for origin discrimination and authentication study of these products (Lange et al., 2019). For example, this technique has been successfully applied for origin discrimination analysis of sesame seeds (Young et al., 2017), rice (Lange et al., 2019), tea leaves (Zhang et al., 2020), wheat (Zhao et al., 2013), potato (Mahne, et al., 2018), tomato (Fragni et al., 2018), onion (Ariyama et al., 2007), salad (Araújo et al., 2019), cabbage (Mahne et al., 2017 and etc.

Commonly, many trace and abundant elements in food are determined using inductively coupled plasma with atomic emission spectrometry/optical emission spectrometry/mass spectrometry (ICP-AES/OES/MS), which allows simultaneous determination of multi-elemental composition (Dion et al., 2008, Ma et al., 2016, Zhang et al., 2018). These tools have been frequently applied for the assessment of various elements in different samples.

Origin discrimination and authentication studies usually involve the interaction of multiple variables and hence, requires application of multivariate statistical tools to analyze such a complex set of data. Different studies utilized different statistical tools such as: principal component analysis (PCA), linear discriminant analysis (LDA), partial least squares discrimination analysis (PLS-DA), orthogonal partial least squares discriminant analysis (OPLS-DA), support vector machines (SVM), random forest classification (RFC), artificial neural networks (ANN). PCA is a dimension-reduction tool that is often used to reduce a large set of variables to a small set. Some studies used PCA as preliminary steps to visualize data and obtain information for further analysis using other statistical tools. For example, PCA was used as a preliminary tool prior to the application of other statistical tools such as LDA, OPLS-DA and SVM during the origin authentication study of rice (Pracha et al., 2013), tea (Zhang et al., 2013), wheat (Zhao et al., 2020), potato (Magdas et al., 2017) and vegetables (Araújo et al., 2019). However, other studies used PCA as an independent data analysis tool for origin authentication of plant crop and herb products. For example, Lange, et al., 2019 used PCA and successful classified origins of rice samples. It was also successfully applied for origin discrimination analysis of sesame seed (Young et al., 2017) and onion (Ariyama et al., 2007). LDA on the other hand was found to be the most effective and frequently used statistical tool by the studies conducted on this area. It is one of the most popular supervised pattern recognition methods that give a classification of groups from a linear combination of independent variables. LDA has been applied for origin authentication study of different plant crops and herb products such as; rice (Lange et al., 2019), tea leave (Xiancai et al., 2018), sesame seed (Young et al., 2016), wheat (Liu et al., 2017), potato (Mahne et al., 2018), onion (Ariyama et al., 2007), and tomato (Fragni et al., 2018). All of these studies have made a successful origin classification (from 89 to 100 %) of these products using LDA.

The present study mainly focused on classifying sesame seeds obtained from the three main producer regions (Gondar, Humera and Wollega) in Ethiopia by employing elemental analysis using ICP-OES and chemometric tools.

## 2 Materials and methods

### 2.1 Sample area and sample collection

**Fig. 1:**
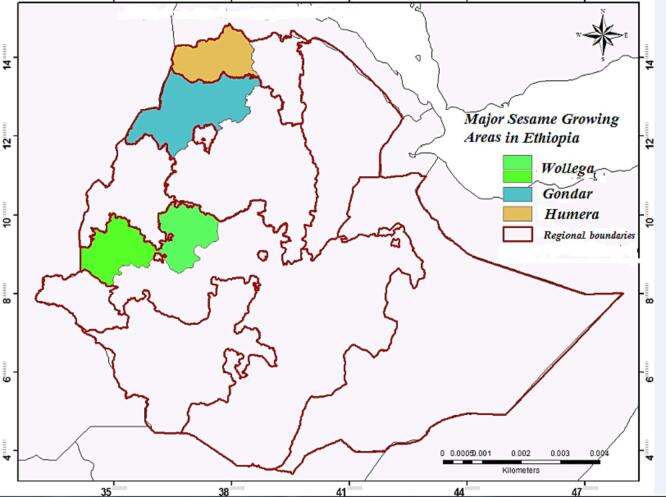
The three sesame seed sampling regions (Humera, Gondar and Wollega) in Ethiopia.

The latitude and longitude of the sampling site were recorded (Fig. 1) using a global positioning system (GPS). A total of 93 samples (34 from Gondar, 31 from Humera and 28 from Wollega) 500 g each were collected from Ethiopian commodity exchange (ECX) local branches located in Humera, Gondar, and Bure. The number of samples for the three regions were determined based on the specific local producing areas (woredas) and variety (Whit, Mixed and Red) of sesame seeds. Geographical origin discrimination analysis was then assessed for these three major sesame growing areas, which contribute almost the entire seed export of Ethiopia to the international market.

### 2.2 Chemicals and equipment

Multi-element working standard concentration of Na, Mg, Cr, Mn, Fe, Cu, Co, Ni, Zn, Cd, As and Pb (CPI international, USA), Nitric Acid (Oxford Lab Fine Chem LLP, India), Grinder, Silica crucibles, Thermostated Oven (HeuerGst corporation Ltd. India), Graphite Furnace (DAIHAN Scientific), ICP-OES (Optima^TM^ 8x00, PerkinElmer, Inc. USA).

### 2.3 Sample preparation

All 93 sesame seed samples were manually separated from any external contaminants and washed with deionized water to remove dust. The samples were then dried (100 °C) for 24 h and ground to powder. Then, dry digestion was performed according to the procedure stated by the food code for trace nutrients test with some modifications (Young et al., 2017). Exactly 1 g of powder sample was taken into a porcelain crucible, and made to ash in a furnace at 600 °C for 4 hrs. The residue (white ash) was dissolved in 10 mL of 0.5-M HNO_3_, filtered (Whatman filter paper No. 5A) into a 100-mL volumetric flask, and filled with deionized water obtained from the reverse osmosis osmosis-deionization system (EVOQUA Water Technologies LLC). All samples were prepared and analyzed in triplicates. Then the mean and their respective standard deviations were calculated.

### 2.4 Method validation

Instrument and method performances such as precision, % recovery, limit of detection (LOD) and limit of quantitation (LOQ) were measured by analyzing blanks, standards and spiked samples with various levels of standards. The recovery test was performed at two different concentrations of spiked samples, at the minimum and maximum level of concentration of the elements detected in the samples. There had been one control sample for every sample sets to be able to determine the change induced by various factors. Then, a six-point calibration curve was established for each element to be determined from their multi-element stock standard solution. The standard calibration concentration ranges of Na, Mg and Fe was between 0.5 and 600 mg/kg and Cr, Mn, Co, Ni, Cu, Zn, Cd, Pd and As was between 0.0001 and 30 mg/kg. Finally, all the samples were analyzed for the level of these elements using ICP-OES.

### 2.5 Statistical analysis

All analyses were conducted in triplicate samples and mean values of multi-element concentrations of sesame seeds were subjected to various chemometric data analysis using STATA, version 14.0 software (1985–2015 Stata Corp LP), SIMCA (Umetrics, Sweden) and SPSS 20 (IBM Corp, USA). The concentration of analyzed elements was employed to construct a geographical origin discrimination model using different statistical tools such as, a one-way analysis of variance (ANOVA), principal component analysis (PCA) and linear discriminant analysis (LDA). ANOVA was first used to find out if there were significant differences in multi-element concentrations among the sesame seeds that originated from the three main seed producer regions (Gondar, Humera and Wollega) in Ethiopia. PCA is a dimensionality-reduction method that is often used to reduce the dimensionality of large data sets, by transforming a large set of variables into a smaller one that still contains most of the information in the large set. Hence, in the present study, PCA was used to visualize the underlying structure of the data being analyzed. Then LDA was performed to create an origin classification model for all sesame seed samples found from the three origins. For the PCA model, pareto scaling was chosen because it provided better model reliability parameter. The score and loading plot were used to interpret the results. Statistical parameters such as R^2^X *cum* (cumulative X variation modeled from the n components), Q^2^ *cum* (cumulative variation of X or Y that can be predicted by n components), and n (number of principal components) were used to evaluate the reliability of the model developed using the multielement data for the three sampling regions.

## 3 Results and discussion

### 3.1 Validation results

The accuracy and precision of the method were determined by calculating the relative standard deviation (RSD) and recovery tests of replicate analysis of control laboratory samples spiked with standards. The recovery test was performed at two (high and low) concentration level of added elements taking into consideration of the natural abundance of elements under the study. LOD and LOQ of the method were determined by analyzing replicate blank samples spiked with multi-element standards following the equations.

and LOQ.where δ is the standard deviation and s is the slope of the regression line for the individual element. (Young et al., 2017).

The % recovery of elements added, limit of detection (LOD), and limit of quantitation (LOQ) of the 12 elements analyzed by inductively coupled Plasma-Optical Emission Spectroscopy (ICP-OES) are summarized in Table 3.

### 3.2 Level of elements determined in the sesame seed samples

Elemental analysis was performed for all 93 sesame seed samples obtained from different areas of the three origins (Gondar, Humera and Wollega) in Ethiopia. The minimum, maximum and mean concentrations of 12 mineral elements in the sesame seed samples are summarized in Table 1. A kernel density estimation showed that the concentrations of all analyzed mineral elements in the samples followed a normal distribution. The mean concentration of Mg in the three origins (420, 498 and 384 mg/kg) was found to be the highest level among all the analyzed elements. On the other hand, the average concentrations of Cd, As and Cr in the three origins were below the standard limit values set by the European commission for any sold food items (1 mg/kg, 2 mg/kg, and 5.0 mg/kg) of Cd, As, and Cr respectively (Letícia et al., 2016). The level of these elements are also below the limit value given by the joint WHO/FAO food standard program codex commitment on contaminants in food (WHO/FAO, 2011). Moreover, the average Pb concentrations were also below 5 mg/kg which was set as a tolerable limit for soil and agricultural products (Letícia et al., 2016). However, some of the Gondar’s sesame seed samples showed a slightly higher Pb concentration level than the standard given by FAO/WHO codex general for contaminants and toxins in foods and WHO Encyclopedia of Environmental Science.

**Table 1:** The minimum (Min), maximum (Max), average (Mean) level of 12 elemental concentrations in mg/kg with standard deviation (SD) determined in sesame seed samples collected from the three regions (Gondar, Humera and Wollega).

Origin	Gondar (n = 34)				Humera (n = 31)				Wollega (n = 28)			
Element	Min	Max	Mean	SD	Min	Max	Mean	SD	Min	Max	Mean	SD
Na|	9.5	27.2	16.7	4.4	2.4	6.1	4.2	1.2	13.4	89.4	36.6	18.6
Mg|	245	517	420	58	416	594	498	46	234	535	384	94
Cr|	1.5	1.8	1.6	0.1	0.0	1.6	0.4	0.2	0.0	0.9	0.1	0.02
Mn|	3.2	5.5	3.8	0.5	0.3	1.3	0.6	0.3	0.3	15	10	3.0
Fe|	7.3	19.2	12.8	3.4	0.9	11.5	5.3	2.6	16	42	25	7.0
Co|	0.1	1.4	1.0	0.3	0.02	1.2	0.2	0.3	0.04	0.7	0.2	0.2
Ni|	1.2	1.7	1.4	0.2	0.1	0.8	0.4	0.2	0.03	5.0	1.0	0.9
Cu|	3.2	4.6	3.8	0.3	0.3	0.9	0.5	0.1	0.4	3.8	2.0	0.8
Zn|	4.3	14.4	10.6	2.1	0.8	3.4	1.6	0.5	1.2	14	7.6	3.2
Cd|	0.09	1.8	0.6	0.2	0.00	0.1	0.04	0.02	0.00	0.1	0.1	0.02
Pb|	0.7	2.6	1.3	0.6	0.01	2.5	49	0.7	0.01	2.5	1.5	0.6
As|	0.2	2.2	1.4	0.3	0.1	1.7	0.3	0.4	0.00	0.8	0.2	0.2

The concentration level of some of the elements (Mg, Fe, Mn and Cu) determined was in good agreement with the levels reported by previous studies performed in Chinese, Korean and Indian sesame seeds (Young et al, 2017). Nearly similar findings were reported for the contents of Mg and Na by a study made on sesame seeds obtained from Congo-Brazzaville (Nzikou et al., 2009).

One-way ANOVA, Bonferroni test confirmed that, most pairs of comparison within each of the three areas were not significantly different at 0.05 level. However, except for Co and Ni, there was a significant difference (p < 0.05) in the concentration of the remaining 10 mineral elements among the three origins of sesame seed samples. The Wollega sesame seed showed the highest mean concentration level of Na, Mn and Fe among the three origins (Wollega > Gondar > Humera) while the Gondar and Humera sesame seeds showed the highest level of Zn and Mg respectively. The level of Cr, Co, Ni, Cu and As in the Gondar sesame seed samples were found to be significantly (p < 0.05) higher than the Humera and Wollega samples while the difference was not significant for those elements concentration between the Humera and Wollega sesame seed. The contents of Mg in the Humera sesame seeds were significantly higher than those in the Gondar or Wollega samples (p < 0.05). The Gondar samples could be distinguished from the Wollega and Humera samples because they had the highest Zn content.

### 3.3 Origin discrimination model

Despite the presence of other contributing sources, natural soil conditions of the sesame seed growing areas is the most important determining factor resulting in great differences in the elemental content. According to the results of one-way ANOVA testing, the concentrations of 10 mineral elements (Na, Mg, Cr, Mn, Fe, Cu, Zn, Cd, Pb and As) were found to be significantly different among the three regions (p < 0.05). Therefore, only these elements were considered for further statistical analysis to discriminate the geographical origin of the sesames seeds growing in those three regions.

Principal component analysis (PCA) is first applied to be able to perform exploratory analysis and obtain an overview of data and patterns in the data so that to transform highly correlating variables into a smaller set of meaningful factors. Hence, to evaluate the grouping nature of the 93 sesame seed samples, the concentrations of 10 mineral elements that showed significant differences by ANOVA test were subjected to PCA, an unsupervised technique. The dataset was Pareto scaled before the construction of the PCA model. PCA is also sensitive to an outlier, so possible suspected data were first examined.

Then from the PCA result, three principal components (PCs) with eigenvalues exceeding one were extracted, which accounted for 70 % (PC1, 34 %, PC2, 27 % and PC3 9 %) of the total variance. All the 10 elements employed were loaded on the three components (PC1, PC2 and PC3). The goodness-of-fit of the PCA model constructed was evaluated in terms of R^2^X (the variation of the dataset explained) and Q^2^ (goodness of prediction of the mode) values. The R^2^X *cum* and Q^2^ *cum* values of the constructed model were found to be 92.1 and 75.2 % respectively. In addition, the value of R^2^X is greater than Q^2^, while the difference between R^2^X and Q^2^ less than 20 % (0.2), which further confirms the model's reliability.

**Fig. 2:**
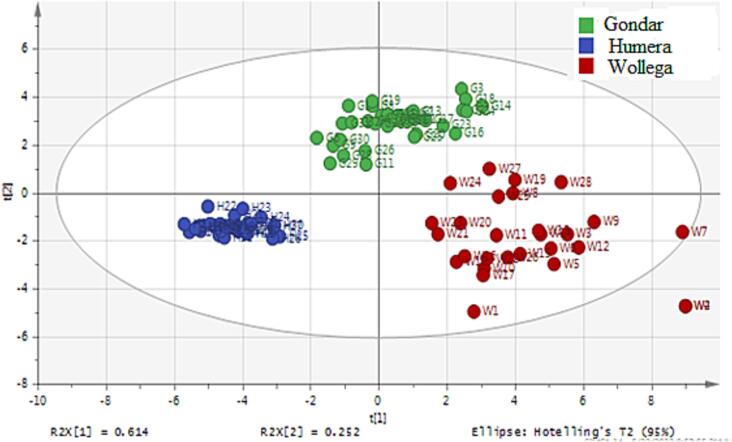
PCA score plot of component one versus component two.

**Fig. 3:**
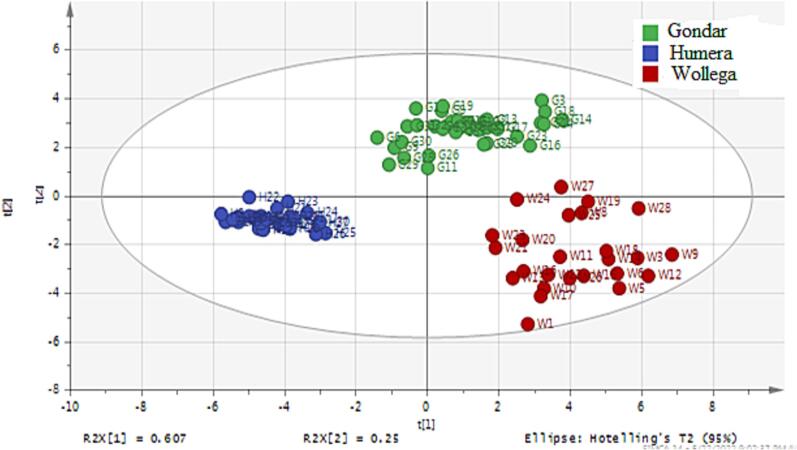
PCA score plot of component one versus component two after excluding two Wollega outlier samples.

The two-dimensional scatter plot of the scores for PC1-PC2 (Fig. 2) showed some trends of clustering with partial overlap of samples from Gondar and Wollega. All the Humera samples were clearly separated from all other samples originating from Gondar and Wollega. As it is also shown in the figure (Fig. 2) three of the Wollega samples were separated from the rest of other samples obtained from the same origin. Hence, the PCA model was rebuilt by excluding two samples (both are found to be outliers) that originated from Wollega. The resulting model (Fig. 3) showed an improved grouping nature of samples based on their respective origins.

Linear discriminant analysis (LDA) was then applied to create an origin classification model for all sesame seed samples obtained from the three areas. LDA is used to describe the differences between groups and to exploit those differences in allocating (classifying) observations of unknown group membership to the groups. LDA reduces dimensions as PCA, but it focuses on maximizing the discrimination among known groups. Hence, in this study, LDA was applied to identify some data which have high predictive ability for the samples. The model was used for 10 analyzed mineral elements based on the previous ANOVA test. Then two discriminant functions were constructed according to the Wilks’ Lambda factor which measures the likelihood nature of groups based on the value 1.0 which denotes zero discriminatory power to 0.0 which demonstrates 100 % discriminatory power. The standardized canonical discriminant function coefficients of all the analyzed elements were:

**Function 1**:.*2583541Na + 0.4387521 Mg -.5056775Cr + 3345452Mn +.0116078Fe -.1810675Zn -.7944334Cu -.2549759Cd + 0.8497046 Pb -.4710599As*

**Function 2**:*.2244367Na - 0.5150917 Mg -.3578092Cr +.3569545Mn +.7071911Fe -.023435Zn +.5406643Cu +.1666604Cd -.0612103Pb +.1432939As*

These two functions explained 100 % of the total variance (function 1 explained 70 % while the rest 30 % of the total variance were accounted for function 2), and the Wilks’ Lambda ratio value for function 1 (canonical correlation = 0.9930) were 0.0134 and function 2 (canonical correlation = 0.9689) were 0.1684. The results showed that there were significant differences (Prob > F = 0.000) of elemental content among the three regions.

**Fig. 4:**
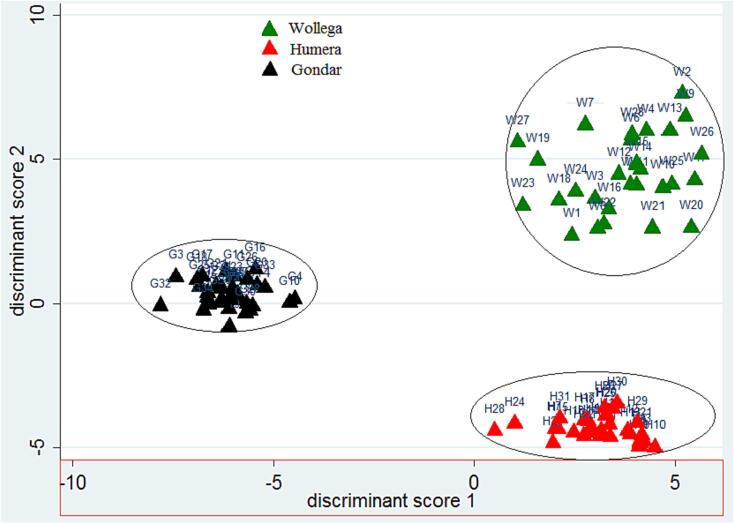
The score scatter plot of function one (82.1%) versus function two (17.9%) of linear discriminant model established for 93 sesame seed samples of Gondar, Humera and Wollega.

The score scatter plot of the two functions (Fig. 4) confirmed that LDA created a better discrimination of sesame seed samples among the three origins. From the figure it appears that all samples concentrated in to three separated distribution area according to their origin. These results indicated that the sesame seed samples from different regions were well distinguished from one another. The leave-one-out cross-validation analysis also confirmed that 100 % of the samples under investigation were correctly classified to their respective geographical origin (Table 2).

**Table 2:** Predictive ability LDA for the geographical origin discrimination analysis of 93 sesame seed samples obtained from Gondar, Humera and Wollega.

Sample origins	Investigated samples	Observed Group Membership			
		Gondar	Humera	Wollega	Correctly Assigned (%)
Gondar	34	34	0	0	100
Humera	31	0	31	0	100
Wollega	28	0	0	28	100
Total	93	34	31	28	100

**Table 3:** Quality control results for elemental analysis of sesame seed by ICP-OES instrument.

Element	R^2^	% Recovery at two concentration level		LOD (mg/kg)	LOQ (mg/kg)	RSD
		Level 1 ± SD	Level 2 ± SD			
Na*	0.999846	112 ± 4.6	99 ± 3.3	0.01	2.5	2.3
Mg*	0.999646	96 ± 3.9	89 ± 3.6	0.008	10	1.8
Cr**	0.998034	85 ± 2.2	86 ± 1.8	0.00005	0.001	4.8
Mn**	0.999813	86 ± 3.7	89 ± 5.5	0.00005	0.1	4.2
Fe*	0.999813	98 ± 6.6	92 ± 4.7	0.1	0.1	3.1
Co**	0.996855	82 ± 4.8	96 ± 4.4	0.00005	0.01	5.6
Ni**	0.996855	87 ± 6.4	93 ± 7.8	0.00005	0.01	4.7
Cu**	0.998034	92 ± 5.2	87 ± 6.3	0.00005	0.1	3.3
Zn**	0.999813	105 ± 7.4	94 ± 8.1	0.008	0.5	2.9
Cd**	0.995846	80 ± 4.1	81 ± 5.7	0.00001	0.5	5.4
Pb**	0.996855	83 ± 3.7	80 ± 8.2	0.00001	0.05	4.5
As**	0.996855	86 ± 4.3	82 ± 7.4	0.00001	0.05	3.8

*Elements calibration range is 0.5–600 mg/kg and recovery concentration for level 1 and 2 is 100 mg/kg and 5 mg/kg respectively, **Elements calibration range is 0.000.1–30 mg/kg and recovery concentration for level 1 and 2 is 5 mg/kg and 0.1 mg/kg respectively.

Hence, multi-element analysis combined with LDA was found to be a useful method for geographical origin discrimination of sesame seeds. In fact, multi-element technique combined with LDA had been applied and proved to be an effective method for the geographical origin determination of various agricultural products. For example, it has been applied for origin authentication study of different plant crop and herb products such as; rice (Pracha et al., 2013), tea leave (Xiancai et al., 2018), sesame seed (Young et al., 2016), wheat (Liu et al., 2017), potato (Mahne et al., 2018), onion (Ariyama et al., 2007) and tomato (Fragni et al., 2018). All of these studies have made a successful origin classification (from 89 to 100 %) of these products with LDA.

Fisher’s linear discrimination functions for the three regions were also generated from LDA model and verified by taking 12 randomly selected sesame seed samples (5 from Gondar, 4 from Humera 3 from wollega). All the samples were first analyzed for 10 elements which were used to create the discriminant functions. Then, geographical origin discrimination was made with respect to the three functions. All predictions were found to be correct for all 12 seed samples in terms of their geographical origins. Hence, the LDA method based on the mineral element fingerprints was found to be suitable for verifying the geographical origins of the sesame seed samples collected from three regions in Ethiopia.

### 3.4 Conclusion

In conclusion the concentration of 12 elements (Na, Mg, Cr, Mn, Fe, Cu, Co, Ni, Zn, Cd, As and Pb) were determined in 93 sesame seed samples obtained from three regions (Gondar, Humera and Wollega) in Ethiopia. The concentration of 10 elements (Na, Mg, Cr, Mn, Fe, Cu, Zn, Cd, As and Pb) were found to be significantly different among the three regions (p < 0.05). Then geographical origin discrimination model was constructed using the concentration of the above 10 elements. The resulting origin classification rate with the LDA model was found to be 100 % for all 93 samples. The constructed LDA model was also verified by taking 12 sesame seed samples (5 from Gondar, 4 from Humera 3 from wollega) and all 12 samples were correctly assigned to their respective origins. Hence, as a result of this study, it can be concluded that, the multi-element analysis combined with LDA were found to be an effective way to discriminate sesame seeds originating from Ethiopia.

## CRediT authorship contribution statement

**Wasihun Abebe Hika:** Methodology, Investigation, Writing – original draft, Formal analysis. **Minaleshewa Atlabachew:** Supervision, Conceptualization, Formal analysis. **Meareg Amare:** Supervision.

## Declaration of Competing Interest

The authors declare that they have no known competing financial interests or personal relationships that could have appeared to influence the work reported in this paper.

## Data Availability

Data will be made available on request.
